# Transferrin-Conjugated Melittin-Loaded L-Arginine-Coated Iron Oxide Nanoparticles for Mitigating Beta-Amyloid Pathology of the 5XFAD Mouse Brain

**DOI:** 10.3390/ijms241914954

**Published:** 2023-10-06

**Authors:** Moonseok Choi, Junghwa Ryu, Huy Duc Vu, Dongsoo Kim, Young-Jin Youn, Min Hui Park, Phuong Tu Huynh, Gyu-Bin Hwang, Sung Won Youn, Yun Ha Jeong

**Affiliations:** 1 Department of Neurodegenerative Diseases Research Group, Korea Brain Research Institute, 61, Cheomdan ro, Dong gu, Daegu 41062, Republic of Korea; moonseok37@kbri.re.kr (M.C.); rapidozzangs@kbri.re.kr (D.K.); yunyj1203@kbri.re.kr (Y.-J.Y.); gyubin2023@kbri.re.kr (G.-B.H.); 2 Department of Radiology, School of Medicine, Daegu Catholic University, 33, Duryugongwon-ro 17-gil, Nam-gu, Daegu 42472, Republic of Korea; rjunghwa@gmail.com (J.R.); duchuy1812@gmail.com (H.D.V.); pmh023@kakao.com (M.H.P.); huynhtuphuong21@gmail.com (P.T.H.)

**Keywords:** Alzheimer’s disease, nanoparticle, melittin, iron oxide, transferrin, amyloid plaque

## Abstract

Alzheimer’s disease (AD) is one of the most prevalent neurodegenerative diseases and a major contributor to dementia. Although the cause of this condition has been identified long ago as aberrant aggregations of amyloid and tau proteins, effective therapies for it remain elusive. The complexities of drug development for AD treatment are often compounded by the impermeable blood–brain barrier and low-yield brain delivery. In addition, the use of high drug concentrations to overcome this challenge may entail side effects. To address these challenges and enhance the precision of delivery into brain regions affected by amyloid aggregation, we proposed a transferrin-conjugated nanoparticle-based drug delivery system. The transferrin-conjugated melittin-loaded L-arginine-coated iron oxide nanoparticles (Tf-MeLioNs) developed in this study successfully mitigated melittin-induced cytotoxicity and hemolysis in the cell culture system. In the 5XFAD mouse brain, Tf-MeLioNs remarkably reduced amyloid plaque accumulation, particularly in the hippocampus. This study suggested Tf-LioNs as a potential drug delivery platform and Tf-MeLioNs as a candidate for therapeutic drug targeting of amyloid plaques in AD. These findings provide a foundation for further exploration and advancement in AD therapeutics.

## 1. Introduction

Alzheimer’s disease (AD) is the most common neurodegenerative disorder and contributes to 60–70% of global dementia cases [1]. With an increase in the worldwide aging population, there has been a rise in the number of patients with AD and care costs of dementia [2]. The number of AD patients is projected to reach approximately 150 million by the year 2050, with approximately one-third of the global population over the age of 65 developing this condition [3]. This escalating burden of AD underscores the urgent need for effective strategies to address the serious impact of this condition on public health [4].

Beneath the clinical presentation of cognitive impairment and behavioral disturbances, AD presents distinctive pathological features, such as amyloid plaques as aggregated β-amyloid (Aβ) in the brain, hyperphosphorylation of tau protein leading to tangles in neurons, and reactivation of glial cells such as astrocytes and microglia [5]. In relation to Aβ and tau loads, microglial-activation-mediated neuroinflammation has been reported to lead to neuronal death and dysfunction in AD [6,7].

Considering that AD development is closely linked to Aβ-accumulation-mediated neuroinflammation and that melittin peptide exerts anti-inflammatory effects, we hypothesized that, as observed in other disorders, the neuroinflammation in AD might also be mitigated by a potent anti-inflammatory drug such as melittin. Bee venom and its derived active components have been used in folk remedies and traditional medicine to relieve pain symptoms and inflammatory diseases [8]. Recently, melittin peptide, the main component of honeybee venom, has gained interest as an anti-neurodegenerative agent, primarily due to its anti-inflammatory functions. Nguyen et al. reported that melittin exerted antioxidant and neuroprotective actions against neural oxidative stress caused by Aβ_1–42_ and further enhanced cognitive function in learning- and memory-deficit mice by dose-dependently promoting neural cell genesis in the hippocampal dentate gyrus (DG) region [9,10].

However, melittin is a cytolytic peptide, and its intravenous administration causes non-specific cell toxicity (such as cell membrane pore formation) and hemolysis of red blood cells [11,12]. To circumvent these challenges, a previous study [13] developed melittin-loaded L-arginine-coated iron oxide nanoparticles (MeLioNs) by loading melittin on the surface of L-arginine-coated iron oxide nanoparticles (LioNs) as the core–shell structure. Iron oxide nanoparticles were selected as the core compartment of this core–shell nano formulation since iron is an essential element of metabolism, and iron oxide nanoparticles have been widely used for drug delivery and as a magnetic resonance imaging contrast agent, with proven safety [14,15]. L-arginine was used to crosslink iron oxide nanoparticles and melittin peptides in anticipation of potential health benefits such as the release of nitric oxide [16].

Furthermore, we hypothesized that transferrin-conjugation would promote melittin delivery across the blood–brain barrier (BBB) through transferrin receptor binding. Conventional AD drugs targeting the underlying pathology, such as amyloid plaque accumulation and tau protein abnormalities, encounter a challenge when they cross the BBB, which is responsible for regulating the passage of substances from the bloodstream into the brain [17]. The restricted permeability of the BBB limits the number of therapeutic agents that can enter the brain in effective concentrations [18]. To address this obstacle of the BBB, transferrin-conjugated nanoparticle-based drug delivery systems aim to enhance BBB penetration [19,20]. Although there have been some studies on transferrin receptors that target BBB penetration [21,22], it is not yet clear whether transferrin-conjugated nanoparticles effectively penetrate through the BBB and deliver the targeted drug to brain lesions.

The goal of this study was to evaluate whether transferrin-conjugated melittin-loaded L-arginine-coated iron oxide nanoparticles (Tf-MeLioNs) demonstrate BBB permeability and regulate amyloid plaques in AD model mice. To achieve this goal, we treated 5XFAD mice with Tf-MeLioNs and investigated the AD-related pathological changes.

## 2. Results

### 2.1. Synthesis and Characterization of Tf-MeLioNs

**Figure 1:**
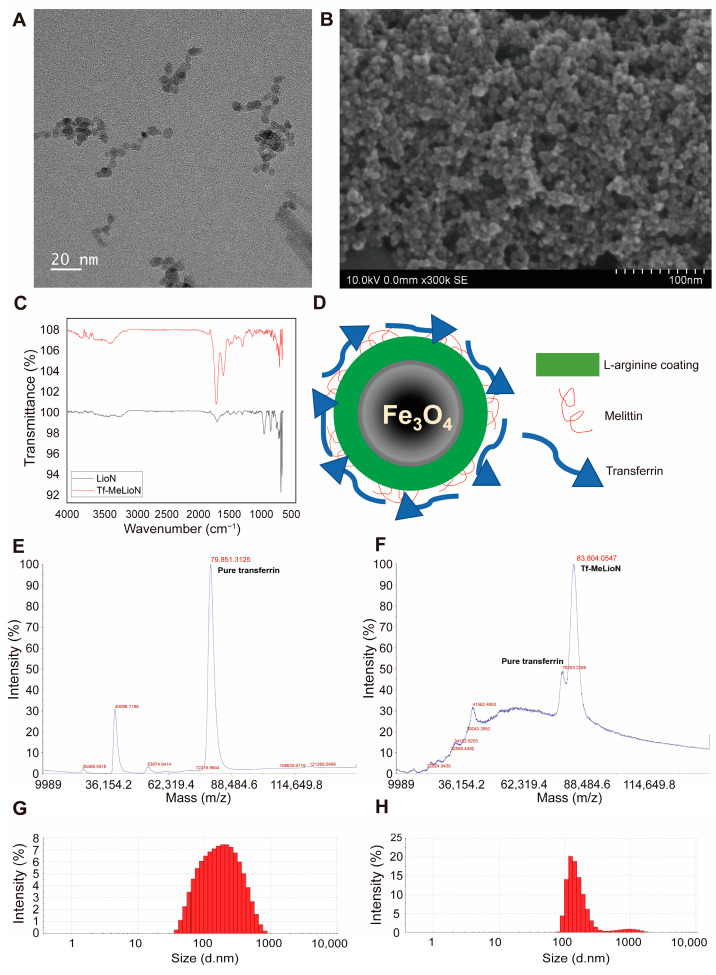
Synthesis and characterization of Tf-MeLioNs. Transmission electron microscopy (**A**), scanning electron microscopy (**B**), and Fourier-transform infrared spectroscopy (**C**) images of the synthesized Tf-MeLioNs. Schematic structure of the Tf-MeLioNs (**D**). Matrix-assisted laser desorption/ionization–time-of-flight spectra of pure transferrin (**E**) and Tf-MeLioNs (**F**). Hydrodynamic size of MeLioNs (**G**) and Tf-MeLioNs (**H**), as analyzed using dynamic light scattering. LioNs, L-arginine-coated iron oxide nanoparticles; Tf-MeLioNs, transferrin-conjugated melittin-loaded LioNs.

Transmission and scanning electron microscopy of Tf-MeLioNs showed a gross spherical core shape (Figure 1A) and well-dispersed nanoparticles, with sizes in the range of 8–12 nm on a grid (Figure 1B). The corresponding bands for each functional group upon Fourier-transform infrared (FT-IR) spectroscopy are shown using black and red lines (Figure 1C). Transferrin-conjugation occurred successfully in the MeLioNs to form Tf-MeLioNs. There were two intense peaks observed between 580 cm^−1^ and 630 cm^−1^, representing the stretching vibrations of metal–oxygen (Fe–O) bonds in the crystalline Fe_3_O_4_ structure. The band at ~1629 cm^−1^ and the broad band at ~3435 cm^−1^ represented the appearance of hydroxy groups in the OH-bending and OH-stretching vibration modes, respectively. In the LioNs spectrum, there was a highly intense broad band in the range of 3310–3350 cm^−1^, which could be attributed to N–H bonds, close to the strong, broad band for O–H bonds in the range of approximately 3030–3570 cm^−1^. This signal (3310–3350 cm^−1^) was more intense in the spectrum of Tf-MeLioNs. An amide bond formed between two functional groups, such as C=O and N–H, which contributed to the increased intensity of the peaks at ~1650 cm^−1^ and ~1600 cm^−1^ in the Tf-MeLioNs spectrum. The schematic structure of Tf-MeLioNs, consisting of a core made of iron oxide nanoparticles coated by an L-arginine layer to function as a surfactant and a crosslinker to link melittin to iron oxide nanoparticles, is given in Figure 1D. Transferrin was conjugated to MeLioNs by means of an amide bond between the carboxyl group of Tf and the amine group of melittin. Data generated using matrix-assisted laser desorption/ionization–time-of-flight (MALDI-TOF) mass spectrometry shows the molecular weight of pure transferrin and Tf-MeLioNs (Figure 1E and Figure 1F, respectively). The molecular weights of pure transferrin and Tf-MeLioNs were recorded as 79,851.3125 and 83,804.0547 Da, respectively. The molecular weight difference between pure transferrin and Tf-MeLioNs represented the mass of MeLioNs, which was calculated as 83,804.0547−79,851.3125 = 3952.7422 Da. The obtained result from the mass spectroscopy data matched well with the theoretical mass of MeLioNs, i.e., 3246 Da, thus confirming the successful conjugation of transferrin to MeLioNs. Lastly, the hydrodynamic size of fully developed Tf-MeLioNs was assessed using dynamic light scattering analysis, which determined the average size of MeLioNs as 142.2 nm (polydispersity index = 0.35) and that of Tf-MeLioNs as 205.9 nm (polydispersity index = 0.53) (Figure 1G,H).

### 2.2. Biosafety Profile of Tf-MeLioNs In Vitro and In Vivo

**Figure 2:**
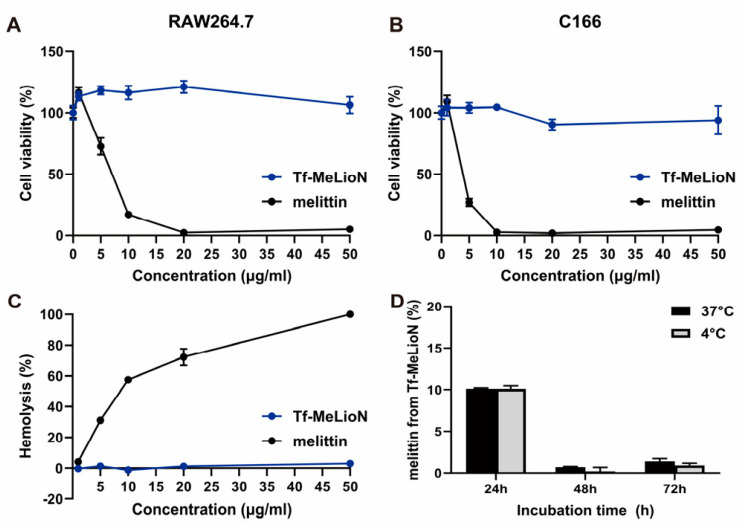
Biosafety profile of Tf-MeLioNs in vitro. Cell viability test of Tf-MeLioNs and free melittin in RAW 264.7 (**A**) and C166 (**B**) cells upon treatment for 24 h. Hemolysis test of Tf-MeLioNs and free melittin in the mouse blood (**C**). In vitro release profiles of melittin from the Tf-MeLioNs at 37 °C and 4 °C (**D**). LioNs, L-arginine-coated iron oxide nanoparticles; Tf-MeLioNs, transferrin-conjugated melittin-loaded LioNs.

RAW 264.7 macrophages and C166 endothelial cells were exposed to free melittin and Tf-MeLioNs at varying concentrations for 24 h (Figure 2A and Figure 2B, respectively). While free melittin exhibited limited and dose-dependent cell viability in RAW 264.7 (116.60 ± 4.11, 72.73 ± 6.73, 17.03 ± 0.69, 2.22 ± 0.17, and 4.95 ± 0.88) and C166 (109.05 ± 5.08, 26.99 ± 3.03, 2.58 ± 0.23, 1.76 ± 0.12, and 4.41 ± 0.21) cells at concentrations of 1, 5, 10, 20, and 50 μg/mL, respectively, Tf-MeLioNs displayed excellent cell viability, even at the higher concentration of 50 μg/mL. The hemolysis assay revealed that free melittin induced dose-dependent hemolysis (4.14 ± 0.37, 31.19 ± 1.45, 57.59 ± 1.48, 72.24 ± 5.41, and 100 ± 0.99) at concentrations of 1, 5, 10, 20, and 50 μg/mL, respectively, while Tf-MeLioNs exhibited minimal hemolytic activity at concentrations up to 20 μg/mL (0–1.23%), and only 3 ± 1.13% hemolysis of mouse red blood cells at the higher concentration of 50 μg/mL (Figure 2C). Released melittin from the Tf-MeLioNs was measured in in vitro drug release tests at 37 °C and 4 °C, over 24, 48, and 72 h, respectively, using the bicinchoninic acid (BCA) assay. Over 24 h, approximately 10% of the melittin was released from the Tf-MeLioNs (10.1 ± 0.13, 37 °C; 10.1 ± 0.39, 4 °C), and it was considered as an initial burst release amount. Over 48 and 72 h, the released melittin from the Tf-MeLioNs was approximately 1% (0.7 ± 0.07, 0.2 ± 0.47; 37 °C/1.4 ± 0.34, 0.9 ± 0.27; 4 °C) (Figure 2D). These results support the notion that the Tf-MeLioNs effectively mitigated melittin-induced cytotoxicity and hemolysis while maintaining a controlled drug release profile.

**Figure 3:**
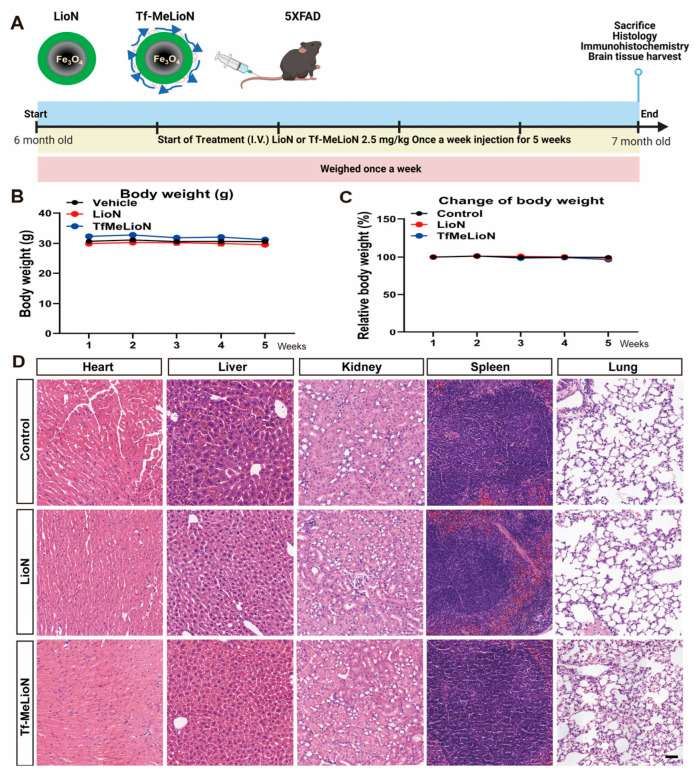
Biosafety profile of Tf-MeLioNs in vivo. Experimental scheme (**A**). The relative body weight change was recorded weekly during the five weeks of Tf-MeLioNs tail vein injection (**B**,**C**). Representative hematoxylin–eosin staining images of the mouse organs (heart, liver, kidney, spleen, and lung) (**D**). LioNs, L-arginine-coated iron oxide nanoparticles; Tf-MeLioNs, transferrin-conjugated melittin-loaded LioNs. Scale bar: 50 μm.

To determine the effect of Tf-MeLioNs treatment on AD progression, Tf-MeLioNs, LioNs, or vehicle (control) were administered via the tail vein (2.5 mg/kg; one injection every week) to male 5XFAD AD transgenic mice aged 24 to 29 weeks. Five weeks after treatment, all the mice groups were sacrificed for histological and molecular analyses. The experimental scheme and timeline are summarized in Figure 3A.

During the 5 weeks, monitoring the body weights of the 5XFAD mice upon administering vehicle (control), LioNs, and Tf-MeLioNs revealed no significant changes across all the groups (Figure 3B,C).

The 5XFAD male mice were sacrificed after the experimental scheme of Tf-MeLioNs tail vein injection. Hematoxylin–eosin staining of the heart, liver, kidney, spleen, and lungs demonstrated the absence of histological alterations, such as inflammatory cell infiltration, necrosis, or fibrotic changes, as compared to that in the untreated control and LioNs-treated mice, thereby affirming the safety of the Tf-MeLioNs (Figure 3D). Consequently, these findings corroborated the absence of toxicity associated with Tf-MeLioNs, not only in terms of cellular toxicity and hemolytic capability but also in the context of in vivo toxicity.

### 2.3. Effects of Tf-MeLioNs Treatment on the Number of Amyloid Plaques in the 5XFAD Mice

To confirm whether the Tf-MeLioNs successfully crossed the BBB and accumulated in the brain, we used the In Vivo Imaging System (IVIS^®^) and Prussian Blue staining (Figure S1A). The distribution of Tf-MeLioNs administered via the tail vein was meticulously monitored at 10 min, 24 h, and 48 h after administration (Figure S1A). At 10 min, Tf-MeLioNs arrived in the brain area with a temporal peak and demonstrated high fluorescence along the injection route of the tail vein as well. Even after 24 h, the Tf-MeLioNs showed high fluorescence. However, after 48 h, no fluorescence was found in the brain, but some renal excretion was observed from the bladder fluorescence. These results suggested that Tf-MeLioNs prominently target the brain shortly after administration (at 10 min) and are gradually cleared by the 48 h interval. Prussian Blue staining of the transgenic (TG) mouse brain showed a blue dot-like staining of iron oxide contained within the Tf-MeLioNs, suggesting that the Tf-MeLioNs arrive in the mouse brain across the BBB (Figure S1B). Notably, Tf-MeLioNs selectively localized within the hippocampal DG, a region associated with memory/cognitive function [23].

**Figure 4:**
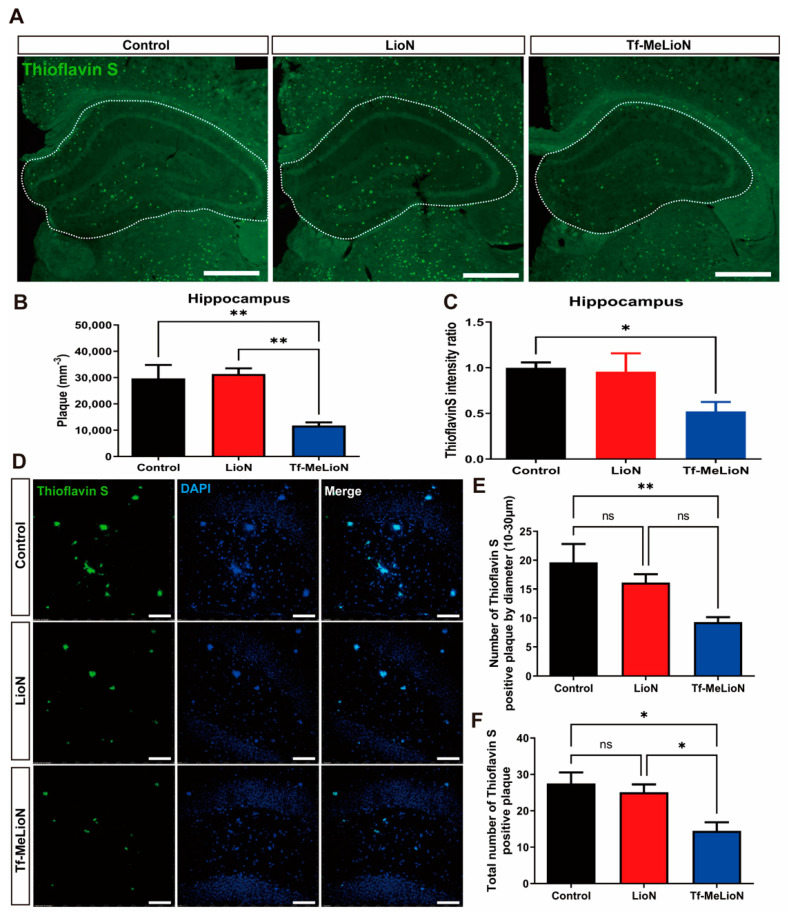
Effects of Tf-MeLioNs treatment on the density of amyloid plaques in the 5XFAD mice. Representative images of Thioflavin S staining of the hippocampus (**A**); scale bar: 1000 μm. Quantification of the number (**B**) and intensity (**C**) of Thioflavin-S-positive plaques in the hippocampus. Representative images of Thioflavin S staining of the hippocampal dentate gyrus (**D**); scale bar: 100 μm. Quantification of the number of Thioflavin-S-positive plaques with a diameter between 10 and 30 μm (**E**) and total number of Thioflavin-S-positive plaques (**F**) in the dentate gyrus. * *p* < 0.05 and ** *p* < 0.01 (one-way analysis of variance with Tukey’s post hoc test). Data are presented as mean ± standard error of the mean. *n* = 4–5 per group for analysis of the number of amyloid plaques. LioNs, L-arginine-coated iron oxide nanoparticles; Tf-MeLioNs, transferrin-conjugated melittin-loaded LioNs.

The hippocampus is an important area for long-term memory formation and cognitive function and is also the most vulnerable area in AD due to the accumulation of the most amyloid plaques [23]. To observe the changes in amyloid plaques, Thioflavin S staining of the hippocampal region was performed and analyzed using Stereo Investigator after imaging with a Panoramic Slide Scan (Figure 4A). The Tf-MeLioNs group (11,771 ± 1211 mm^−3^) had significantly reduced number of observed plaques [F (2, 10) = 13.61; *p* < 0.01] than the control (29,700 ± 5109 mm^−3^; ** *p* < 0.01) and LioNs (31,338 ± 2171 mm^−3^; ** *p* < 0.01) groups (Figure 4B). In addition, the Tf-MeLioNs group (0.52 ± 0.10) had significantly reduced intensity of Thioflavin-S-positive plaques [F (2, 9) = 5.59; *p* < 0.05] than the control group (1.00 ± 0.06; ** *p* < 0.01) (Figure 4C). These results suggested that the Tf-MeLioNs demonstrate an ability to reduce the number of amyloid plaques within the hippocampus.

To observe the difference in the number of amyloid plaques by diameter between the groups, Thioflavin S staining was performed and analyzed using IMARIS software (version 9.9.1) after imaging of the hippocampal DG region with a confocal microscope (Figure 4D). The Tf-MeLioNs group (9.3 ± 0.86; ** *p* < 0.01) displayed a significantly reduced number of plaques with a 10–30 μm diameter [F (2, 10) = 7.637; *p* < 0.01] than the control group (19.63 ± 3.20) (Figure 4E). The Tf-MeLioNs group (14.50 ± 2.35) also displayed a significantly reduced number of Thioflavin-S-positive plaques with diameters >10 μm [F (2, 10) = 8.006; *p* < 0.01] than the control (27.50 ± 3.05; * *p* < 0.05) and LioNs (25.13 ± 2.12; * *p* < 0.05) groups (Figure 4F). The inhibitory effect of Tf-MeLioNs on amyloid plaque accumulation was validated both in the DG and the entire hippocampus. Notably, the Tf-MeLioNs group displayed a significant reduction in amyloid plaques with diameters in the range of 10–30 μm. Remarkably, plaque accumulation in the 5XFAD mice brain was manifested from an early age of 2 months [24]. Taking this into consideration, we subsequently administrated LioNs and Tf-MeLioNs to 5XFAD mice at an early stage (from 3 months onwards), using the protocol described above. The mice showed no changes in body weight (Figure S2A,B). Moreover, in these early-stage 5XFAD mice, the Tf-MeLioNs group exhibited a substantial reduction in the number of amyloid plaques within the hippocampus than the LioNs group (Figure S2C,D). These results suggested the capacity of the Tf-MeLioNs to inhibit amyloid plaque accumulation, particularly within the hippocampus.

### 2.4. Microglial Activation in the Hippocampal DG upon Tf-MeLioNs Treatment of 5XFAD Mice

We established the inhibitory impact of the Tf-MeLioNs on amyloid plaques within the hippocampus. Therefore, in the next step, we assessed whether the effectiveness of Tf-MeLioNs extended to an amelioration in the activity of microglia, a pathological hallmark associated with AD [25], by assessing the utilization of the microglial marker ionized calcium-binding adapter molecule 1 (Iba1) and changes in cluster of differentiation 68 (CD68; an indicator of microglial activation) via immunofluorescence staining [26].

**Figure 5:**
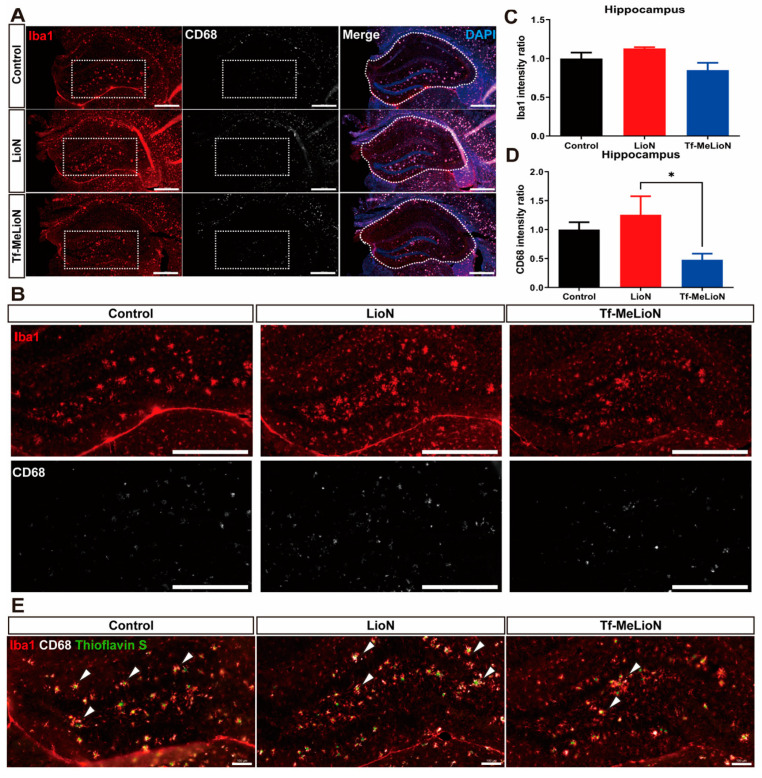
Analysis of microglial activation in the hippocampus upon Tf-MeLioNs treatment. Representative immunofluorescence images of Iba1 and CD68 staining in the hippocampus of male mice; scale bar: 500 μm (**A**). Representative 3× zoom images of Panel A (dashed rectangle); scale bar: 500 μm (**B**). Analysis of the staining intensity for Iba1 and CD68 in the entire hippocampus (**C**,**D**). Representative immunofluorescence images of combined staining for Iba1, CD68, and Thioflavin S in the hippocampal dentate gyrus region showed high expression of Iba1 co-localized with CD68 near the Thioflavin-S-positive plaque (white arrow) (**E**); scale bar: 100 μm. * *p* < 0.05 (one-way analysis of variance with Tukey’s post hoc analysis). Data are presented as mean ± standard error of the mean; *n* = 4–5 per group. LioNs, L-arginine-coated iron oxide nanoparticles; Tf-MeLioNs, transferrin-conjugated melittin-loaded LioNs; Iba1, ionized calcium-binding adaptor molecule 1; CD68, cluster of differentiation 68.

To observe the changes in the activity of microglia, Iba1 and CD68 staining was performed, following which the hippocampal region was imaged (Figure 5A,B). The intensity ratio of Iba1 in the Tf-MeLioNs group (0.85 ± 0.09) was reduced but not significantly different [F (2, 9) = 2.705; *p* = 0.12] than those in the control (1.00 ± 0.08) and LioNs (1.13 ± 0.02) groups (Figure 5C). On the other hand, the intensity ratio of CD68 in the Tf-MeLioNs group (0.48 ± 0.11) was significantly reduced [F (2, 9) = 5.586; *p* < 0.05] as compared to that in the LioNs group (1.26 ± 0.32; * *p* < 0.05), but not the control group (1.00 ± 0.13) (Figure 5D).

Upon combined staining for Thioflavin S, Iba1, and CD68, an evident exaggeration of microglial activity was observed in close proximity to the amyloid plaques. Notably, CD68 expression also displayed an increasing trend within microglia adjacent to amyloid plaques. Additionally, co-localization was noted between CD68- and Iba1-positive microglia in most hippocampal regions (Figure 5E, white arrow). The expression of CD68 alterations in microglia neighboring amyloid plaques necessitates further in-depth investigation and analysis.

Taken together, these results suggested the potent impact of Tf-MeLioNs on diminishing microglial activity within the hippocampus, particularly expression of CD68 within the hippocampal DG region.

### 2.5. Effect of Tf-MeLioNs on Proteins Related to Amyloid Regulation in the Hippocampus

**Figure 6:**
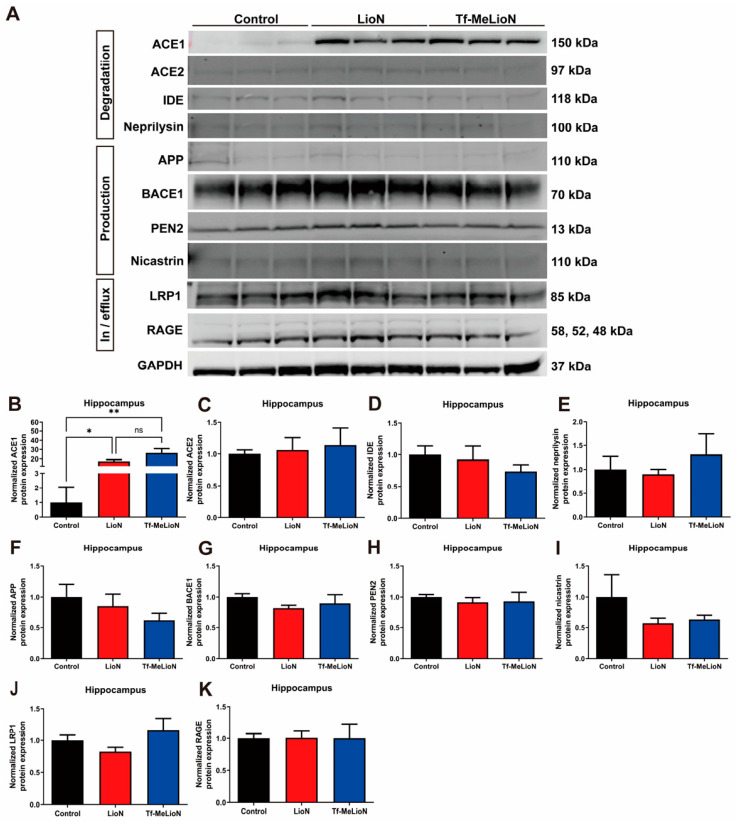
Mechanism of β-amyloid regulation in the hippocampus. Representative Western blot images of the hippocampal proteins (**A**). Analysis of the expression of proteins involved in amyloid degradation (**B**–**E**) and production (**F**–**I**) in the hippocampus. Analysis of the expression of proteins involved in amyloid influx or efflux in the hippocampus (**J**,**K**). * *p* < 0.05, and ** *p* < 0.01, (one-way analysis of variance with Tukey’s post hoc test). Data are presented as mean ± standard error of the mean; *n* = 8–10 per group. LioNs, L-arginine-coated iron oxide nanoparticles; Tf-MeLioNs, transferrin-conjugated melittin-loaded LioNs; ACE1, angiotensin-converting enzyme1; ACE2, angiotensin-converting enzyme2; IDE, insulin-degrading enzyme; APP, amyloid precursor protein; BACE, β-secretase; PEN, presenilin enhancer; LRP, low-density lipoprotein receptor-related protein; RAGE, receptor for advanced glycation end products; GAPDH, glyceraldehyde 3-phosphate dehydrogenase; kDa, kilodalton.

Aβ, a pivotal pathological hallmark of AD, can be influenced by diverse factors that regulate its concentration within the brain [27]. Multiple proteins responsible for Aβ production, degradation, efflux, or influx are recognized contributors [28,29,30,31,32,33]. The potential mechanism underlying the ability of Tf-MeLioNs to reduce the number of amyloid plaques may involve alterations in protein expression. To investigate this, proteins were extracted from the hippocampus of mice subjected to Tf-MeLioNs administration, and the changes in the expression levels of these proteins were assessed using Western blot (Figure 6A).

Interestingly, diverse mechanisms govern the degradation of Aβ. We analyzed the protein expression of representative contributors, such as angiotensin-converting enzyme1 (ACE1), ACE2, insulin-degrading enzyme (IDE), and neprilysin. Notably, as compared to the control (1.00 ± 1.05) group, both the LioNs (17.29 ± 1.88; * *p* < 0.05) and Tf-MeLioNs (26.23 ± 4.62; ** *p* < 0.01) groups displayed significantly increased ACE1 protein expression [F (2, 6) = 18.87; *p* < 0.01] in the hippocampus (Figure 6B). While the Tf-MeLioNs group displayed elevated protein expression of ACE2 and neprilysin as well, the changes did not reach statistical significance (Figure 6C and Figure 6D, respectively). The expression of IDE also showed no significant changes among the groups (Figure 6E). Upon exploring the Aβ production mechanisms encompassing amyloid precursor protein (APP), β-secretase 1 (BACE1), presenilin enhancer 2 (PEN2), and nicastrin, the Tf-MeLioNs group displayed a non-significant decrease in APP and nicastrin protein expression, as compared to the control group (Figure 6F,G), while no discernible variations were noted in the expression levels of BACE1 and PEN2 (Figure 6H and Figure 6I, respectively). Further analysis of Aβ-controlling proteins highlighted that the expression of low-density lipoprotein receptor-related protein 1 (LRP1), a crucial factor for Aβ efflux from the central to peripheral nervous system, showed a tendency to increase in the Tf-MeLioNs group, albeit without statistical significance (Figure 6J). Moreover, no substantial differences were detected in the expression of the receptor for advanced glycation end products (RAGE), a crucial factor for Aβ influx from the peripheral to central nervous system (Figure 6K).

These results suggested that LioNs possess the capacity to modulate the expression of ACE1, a key player in Aβ degradation. Notably, the pronounced alteration in ACE1 in the Tf-MeLioNs group, coupled with changes in neprilysin (which is essential for degradation), as well as APP and nicastrin (which are vital for production), collectively contribute to the reduction in Aβ accumulation. The observed trends in LRP1 expression, which is pivotal for excretion, may drive the synergistic effect on the mechanisms that lead to Aβ reduction.

## 3. Discussion

This proof-of-concept study aimed to evaluate the feasibility of transferrin-conjugation on the surface of cargo nanoparticles and successfully delivered Tf-MeLioNs across the BBB and into the 5XFAD mouse brain, as confirmed using IVIS^®^ and Prussian Blue staining. This study showed that the transferrin-conjugation platform has the potential to overcome ineffective delivery across the BBB, a challenge faced by the current AD treatment.

Beyond symptomatic management, antibody-based drugs, such as aducanumab, gantenerumab, BAN2401, and lecanemab, have shown the potential to target amyloid plaques and modify disease progression. However, their toxicity at high concentrations presents clinical and economic challenges [34,35,36]. To circumvent these, a paradigm shift has emerged towards employing nanoparticles as carriers for drugs, thus revolutionizing AD treatment strategies. For example, incorporating antibody-based drugs within nanoparticles might enhance their BBB permeability, resulting in diminished amyloid plaque aggregation or production within the brain [37]. Nanoparticle delivery systems engineered to target lesion sites stand to impact not only AD but also diverse neurodegenerative disease conditions originating from the abnormality of protein aggregation, such as amyloid plaques, hyperphosphorylated tau, and other associated pathogenic factors in the brain. Transferrin-conjugated platforms can be used to solve the BBB permeability issue of antibody-based drugs. Our study suggested that Tf-LioNs, as well as Tf-MeLioNs, could serve as potential transporters targeting amyloid plaques, thereby offering new possibilities for treatment approaches. Based on this study, Tf-LioNs could be used to enhance the delivery efficiency of various low-concentration AD drug candidates to amyloid lesions across the BBB while mitigating side effects.

In this study, Tf-MeLioNs were designed to target the accumulation of amyloid plaques, particularly in the hippocampus of the brain. The brain delivery efficacy of Tf-MeLioNs was evidenced by its specific distribution within the hippocampal DG region, as observed by means of Prussian Blue staining. However, there is a need for cautious interpretation to ascertain whether this outcome is a result of transferrin-mediated effects. Therefore, further investigations involving the validation of brain delivery mechanisms through blocking of the transferrin receptor are warranted as subsequent studies.

Tf-MeLioNs demonstrated no cytotoxicity or hemolysis at an effective concentration in the range of 10–20 μg/mL, the concentrations utilized for intravenous administration. These results highlight Tf-MeLioNs as a promising approach for enhancing the therapeutic application of melittin, where its potent properties can be harnessed without compromising on safety. There is a need for further investigations to explore the full potential of Tf-MeLioNs as a safe and efficient drug delivery platform.

In addition to delivery, the findings indicated a notable reduction in amyloid plaques within the hippocampal region. Tf-MeLioNs reduced the density and intensity of hippocampal amyloid plaques, as suggested by means of Thioflavin S staining. This effectiveness was consistent in the 10–30 μm-sized amyloid plaques, as well as larger-sized ones. This is the first study to report the action of melittin in reducing the amyloid plaque burden in the AD mouse brain. This finding is in accordance with a recent study that melittin is reported to mitigate cognitive decline by means of its anti-inflammatory action. Nguyen et al. reported that melittin exerts antioxidant and neuroprotective actions against neural oxidative stress caused by Aβ_1–42_ and further enhances cognitive function in learning- and memory-deficit mice by promoting neural cell genesis in the hippocampal DG region in a dose-dependent manner [9,10]. In our study, the effects of Tf-MeLioNs in reducing amyloid plaques within the mouse hippocampus were excellent. Nonetheless, we did not conduct a cognitive test of Tf-MeLioNs-treated 5XFAD mice and could not evaluate whether amyloid reduction by Tf-MeLioNs contributes to the improvements in working or long-term memory. Subsequent investigations are necessary to determine whether this amyloid reduction has the potential to improve cognitive function.

The mechanism of reduced amyloid plaque burden might be related to immunomodulation, and to investigate this, we further evaluated microglial activation markers. Since the activation of astrocytes and microglia is a pathological hallmark in AD [38,39,40,41,42,43], this study examined the relationship between amyloid plaque accumulation and microglial overactivation in the 5XFAD mice brains [44,45]. The microglial markers were found to be localized in the neighborhood of amyloid plaques, suggesting the contribution of amyloid plaque-related neurotoxicity in microglial overactivation and other neuroinflammation [46]. This activation may start a cascade in the secretion of cytokines that contribute to brain inflammation, potentially fueling a feedback loop for Aβ accumulation [47].

Numerous studies have aimed to reduce neuroinflammatory responses within the brain as a therapeutic strategy for AD [48]. In this study, we analyzed the impact of Tf-MeLioNs treatment on microglial reactivation. Assessment of the intensity of Iba1 fluorescence expression using immunofluorescence staining revealed no significant differences. However, the increased expression of CD68 during the phase of microglial reactivation suggests the potential of Tf-MeLioNs to address neuroinflammation. There is a need for further validation of the ability of Tf-MeLioNs to regulate neuroinflammatory responses through analysis of various cytokines secreted by reactivated microglia or exploration of the underlying activation mechanisms. Through such validations, it can be determined whether the anti-inflammatory effects attributed to melittin are also demonstrated in Tf-MeLioNs.

The intricate regulation of Aβ levels within the brain involves the interplay of production, degradation, and transport mechanisms [49,50]. The efficacy of degradation proteins in countering Aβ-related effects is subject to diverse influences involving substrate competition, cellular localization, ion concentrations, and interactions with other pathways [51]. Degradation of Aβ by these proteins, individually or in concert, serves to counteract detrimental processes, including oligomerization, fibril formation, aggregation, and cytotoxicity [52]. Several proteins have been identified to participate in the degradation of Aβ. ACE1, positioned at the N-terminus of the Aβ peptide, degrades residues 16 and 17 [53], while ACE2 targets residues 7, 16, and 17 [54]. IDE exhibits degradation activity toward residues 4 and 8, while neprilysin acts upon positions 16-17, akin to ACE1 [55,56]. In addition, matrix metalloproteinases have also been implicated in Aβ degradation [57].

While our study shows that the elevated ACE1 expression induced by LioNs and Tf-MeLioNs has the potential to enhance Aβ degradation, other studies suggest that ACE1 might exacerbate AD pathology [58]. The propensity of ACE1 to elevate blood pressure in the cerebral vasculature introduces the risk of affected cerebral circulation, oxidative stress, and inflammation. These factors collectively contribute to an increased risk for AD, as indicated by clinical investigations [59,60]. There is a need for comprehensive investigations to uncover the broader implications of ACE1, considering its multifaceted impact on both Aβ metabolism and cerebrovascular dynamics. This understanding holds the key to unraveling the intricate balance between Aβ regulation, vascular health, and AD pathogenesis.

The intricate role of various ions in amyloid plaque aggregation is known. Iron ions emerge as pivotal contributors to this process, with their involvement spanning cellular energy metabolism, oxygen transport, and cellular homeostasis [61]. Dysregulated iron ion concentrations within cells induce oxidative stress, marked by heightened levels of reactive oxygen species, a recognized hallmark of AD [62]. Direct interactions between iron ions and Aβ peptides have been reported, underpinning amyloid plaque formation [63]. Iron ions exhibit the capacity to accelerate the aggregation of Aβ into fibrillary structures, which is characteristic of plaques. This phenomenon is accompanied by the promotion of Aβ oligomerization and an augmentation in fibril quantity; these fibrils are associated with heightened cytotoxicity to increased amyloid aggregation, resulting in cellular membrane damage, promoted permeability, and elevated reactive oxygen species generation [64,65]. However, other investigations have yielded evidence of iron ions engaging with Aβ to inhibit plaque aggregation, forming multimeric complexes [66]. Collectively, these studies underscore the dual nature of iron ions, which can either encourage or interrupt amyloid aggregation and accumulation. The precise intricacies underlying iron ion metabolism in the context of amyloid pathology in AD remain a subject that needs further extensive research.

This study had some limitations. First, although our findings indicated an increase in ACE1 expression, a crucial factor in Aβ degradation, the LioN group also exhibited elevated ACE1 expression without concurrent reduction in amyloid plaques. This discrepancy suggests the existence of mechanisms beyond those explored in our experiments, encompassing amyloid production, degradation, influx, and efflux. Second, we were unable to ascertain the precise mechanism through which melittin from Tf-MeLioN’s content reduces amyloid plaque aggregation. As such, future research should focus on elucidating the pathways underlying the reducing effect on amyloid plaques by Tf-MeLioNs to broaden our understanding of its therapeutic potential.

## 4. Conclusions

This study demonstrated that Tf-MeLioNs have the potential to prevent AD development by reducing the Aβ burden in the hippocampus of 5XFAD mice. Tf-MeLioNs successfully mitigated the melittin-induced cytotoxicity and hemolysis in an in vitro cell culture. In the 5XFAD mouse brain, Tf-MeLioNs were delivered across the BBB and remarkably reduced amyloid plaque accumulation, particularly in the hippocampus. This study suggested the potential of Tf-LioNs as a delivery platform and that of Tf-MeLioNs for ameliorating amyloid-related pathologies in AD and provides a foundation for the use of Tf-MeLioNs as a promising AD treatment strategy.

## 5. Materials and Methods

### 5.1. Animal Experiments and Sampling

The 5XFAD AD model mice were obtained from Jackson Laboratories (MMRC no. 034840) and had the B6SJL-Tg(APPSwFlLon,PSEN1*M146L*L286V)6799Vas/Mmjax strain. These mice were generated by crossbreeding male hemizygous 5XFAD mice with female B6SJLF1 hybrid mice. Male mice aged 5 months were chosen for the experiments. The mice were housed in cages (3–5 mice per cage) and given unrestricted access to food and water. They were kept in a temperature-controlled environment maintained at a humidity of 55 ± 5% under a 12 h–12 h light–dark cycle. For administration of drugs, all mice in the groups were group-housed and weighed once every week. Based on their weight, the mice were injected with the drug via the tail vein at a dose of 2.5 mg/kg once every week. Following drug treatment, the mice were intraperitoneally injected with 2,2,2-tribromoethanol (150 mg/kg, Avertin, Sigma, St. Louis, MO, USA) to induce anesthesia. Serial perfusion was carried out on the mice with 1× phosphate-buffered saline (PBS, pH 7.2) for 5 min, and the mice were fixed in 4% paraformaldehyde. Brain tissue and organs such as the heart, lung, liver, and spleen were collected from the mice after fixation.

### 5.2. Synthesis of Tf-MeLioNs

The synthesis of MeLioNs was conducted according to a previously described method [13]. LioNs were synthesized by means of co-precipitation of ferric and ferrous chloride salts in a molar ratio of 2:1 in an alkaline environment stabilized by L-arginine. FeCl_2_.4H_2_O (0.199 g) and FeCl_3_.6H_2_O (0.541 g) were dissolved in 20 mL of ultrapure water and mixed well with 20 mL of 0.07% L-arginine solution. After the solution temperature reached 80 °C, 7 mL of 25% NH_4_OH solution was added dropwise into the reaction vessel, with vigorous stirring at 1000 rpm, for 1 h until complete precipitation. LioNs were then rinsed three times with water and ethanol, by decantation with a magnet, to remove the residual salts and uncoated L-arginine. The LioNs sample was dried in an oven for 24 h at 60 °C. To load melittin on LioNs (MeLioNs), 1 mL of 1 mg/mL melittin solution was mixed with 1 mL of 2.5 mg/mL LioNs suspension and agitated in a 15 mL tube for 48 h at 4 °C. After the complete loading process, the sample was made to stand 10 min before purification using a 10 kDa centrifugal filter (14,000 rpm, 5 min, Amicon, Darmstadt, Germany). The sample was rinsed three times with ultrapure water and reconstituted in 2 mL of water for later use. For the transferrin conjugation, a mixture of ethyl(dimethylaminopropyl) carbodiimide (0.5 mg)/N-Hydroxysuccinimide (0.25 mg) was added to 0.5 mL of 1 mg/mL transferrin solution and stirred for 30 min, to activate the carboxyl groups of transferrin for conjugation with the amine groups of MeLioNs. Subsequently, 0.5 mL of MeLioNs suspension (0.3 mg/mL melittin; 1.25 mg/mL LioNs) was centrifuged to remove water and redispersed in the coupling buffer (0.5 mL of pH 5.5 2-Morpholinoethanesulphonic acid). The reaction for conjugation lasted for 2 h at room temperature. Finally, the Tf-MeLioNs were purified with Amicon Ultra-4 centrifugal filters (100 kDa) and redispersed into 0.5 mL water.

### 5.3. Transmission and Scanning Electron Microscopy Imaging

A volume of 100 µL as-synthesized Tf-MeLioNs was diluted eight times in absolute ethanol and sonicated for 10 min. Following that, a drop of diluted sample was placed on a 400-mesh copper grid, and the solvent was allowed to evaporate overnight in a 60 °C oven. The sample-containing grid was then subjected to a TEM instrument (field-emission transmission electron microscope; JEM-2100F, JEOL Laboratories, Tokyo, Japan) at 200 kV and a SEM instrument (field-emission scanning electron microscope; HI-9116-0002, Hitachi, Chiyoda-ku, Tokyo, Japan) at 5 kV, handled by an expert at the Korea Basic Science Institute.

### 5.4. FT-IR Spectroscopy

The as-synthesized Tf-MeLioNs solution was freeze-dried to obtain a solid-state sample, which was then analyzed using FT–UV–VIS–IR Spectroscopic Imaging Microscope Vertex 80 (BRUKER, Billerica, MA, USA) in the wavelength range of 600–4000 cm^–1^, by means of the attenuated total reflection method.

### 5.5. MALDI-TOF Spectrometry

The molecular weight of the Tf-MeLioNs sample was analyzed using a MALDI-TOF/TOFTM 5800 system (AB SCIEX, Framingham, MA, USA), with the following operating parameters: operating mode = mass spectroscopy linear mode (positive); mass range (*m*/*z*) = 10–140 kDa; matrix (concentration and solution) = sinapinic acid, 10 mg/mL (0.1% trifluoroacetic acid/30% acetonitrile); bovine serum albumin (BSA) preparation = matrix/sample (29/1); sample preparation = matrix/sample (5/1); data processing = baseline correction, noise filter/smooth, and mass calibration.

### 5.6. Cell Culture (RAW 264.7 and C166)

The murine RAW 264.7 (macrophage) and C166 (endothelial) cells were obtained from American Type Culture Collection (Manassas, VA, USA). The cells were cultured in Dulbecco’s Modified Eagle’s Medium (HyClone, Logan, UT, USA) supplemented with 10% fetal bovine serum (Gibco, Waltham, MA, USA) and 1% penicillin–streptomycin (Gibco). The cells were incubated at 37 °C with 5% CO_2_ until they were 70–80% confluent. For subculture, RAW 264.7 cells were washed with culture medium and harvested using cell scraper, while C166 cells were washed with PBS and detached using 0.25% (*w*/*v*) trypsin/0.53 mM EDTA solution. The cell suspensions were centrifuged at 200× *g* for 5 min.

### 5.7. Cytotoxicity Assay

The cell viability of RAW 264.7 and C166 cells was determined using Cell Counting Kit-8 assay (EZ-Cytox Cell Viability Assay Kit, DAEILLAB, Seoul, Republic of Korea). The cells were seeded into a 96-well culture plate (at a density of 2 × 10^4^ cells) and allowed to attach to the surface of a 96-well plate overnight at 37 °C. The cells were treated with free melittin (1, 5, 10, 20, and 50 µg/mL) and Tf-MeLioNs (equivalent dosages) for 24 h at 37 °C in an atmosphere containing 5% CO_2_. After treatment, 10 µL of CCK-8 reagent was added to each well, and the cells were incubated for an additional 1 h at 37 °C. The cell viability values were then measured in terms of the absorbance at the wavelength of 450 nm using a microplate reader (Molecular Devices, San Jose, CA, USA). Each experiment was carried out in triplicate. The relative cell viability (%) was determined by normalizing the absorbance of the test sample to that of the control sample, as given below:

$$Cell\;viability\left(\%\right)=\left[\frac{A_{sample}-A_{blank}}{A_{control}-A_{blank}}\right]$$

where A_sample_, A_control_, and A_blank_ are the absorbance values of the test, control, and blank samples, respectively.

### 5.8. Hemolytic Activity Assay

To measure hemolytic activity, mouse blood was treated with Tf-MeLioNs and free melittin, according to the protocol described in a previous study [13]. Mouse red blood cells were collected and washed with PBS, and the supernatant was cleared by centrifugation at 1000× *g* for 5 min. Blood (500 µL) was taken from the bottom of the tube and added to 13.5 mL of PBS. Using PBS as blank and Triton™ X-100 as the positive control, either Tf-MeLioNs and free melittin (1, 5, 10, 20, and 50 µg/mL) were added into 200 µL of diluted blood in a 96-well plate. The plates were incubated for 1 h at 37 °C. Following that, the mixture was centrifuged at 400× *g* for 10 min, the supernatant (100 µL) was transferred to a 96-well plate, and its optical density at the wavelength of 414 nm was detected using a plate reader (Molecular Devices). The percentage of hemolysis was calculated as follows:

$$Hemolysis(\%)=\left[\frac{A_{sample}-A_{PBS}}{A_{triton}-A_{PBS}}\right]\times 100$$

where A_sample_, A_triton_, and A_PBS_ are the absorbance values of the sample, Triton™ X-100-treated sample, and 1× PBS, respectively. To determine the zero and 100% hemolysis values, PBS and 0.05% Triton™ X-100, respectively, were added to the red blood cells.

### 5.9. Drug Release Test

The protocol for the drug release test has been described previously [13]. Tf-MeLioNs were dissolved in distilled water, and the supernatant was collected at the given time points (24, 48, and 72 h) at different temperatures, 37 °C and 4 °C. For collecting melittin, the solution was centrifuged in Amicon Ultra-4 centrifugal filter tubes (100 kDa) for 10 min at 10,000× *g*. The amount of melittin released was determined at the wavelength of 562 nm using a microplate reader (Molecular Devices) based on a standard calibration curve of BSA.

### 5.10. Tissue Hematoxylin–Eosin and Prussian Blue Staining

To conduct drug toxicity analyses, hematoxylin–eosin staining was performed on the heart, liver, kidney, spleen, and lungs by the Pathology Department of Daegu Catholic Medical Center (Daegu, Republic of Korea). To observe the distribution of iron oxide nanoparticles, Prussian Blue staining was performed on the brain by the Pathology Department of Daegu Catholic Medical Center. The stained samples were imaged using Panoramic Slide Scan (3dHistech, Budapest, Hungary).

### 5.11. In Vivo Tf-MeLioNs Tracking

The distribution and clearance kinetics of Tf-MeLioNs were obtained using a Bioluminescence and Fluorescence Imaging System (IVIS^®^ Lumina III, PerkinElmer, Waltham, MA, USA). The mice were anesthetized with isoflurane: 3.5% induction for 4 min and 1.5–2.0% for maintenance. Tf-MeLioNs with AF_750_ fluorescence were injected through the tail vein before imaging at 10 min, 24 h, and 48 h after injection (Alexa™ Fluor 750, Thermo Fisher Scientific, Waltham, MA, USA).

### 5.12. Thioflavin S Staining

To analyze amyloid plaques, brain tissues were stained with Thioflavin S solution using a staining protocol described in a previous study [67]. Briefly, brain sections were washed with 1× PBS and then incubated with a 1% Thioflavin S solution (50% ethanol in 1× PBS) for 8 min at room temperature. The stained samples were then washed twice with 85% ethanol for 5 min, 95% EtOH for 5 min, and finally, thrice with 1× PBS. The stained brain tissue samples were imaged using Panoramic Slide Scan, and the number of amyloid plaques was analyzed using Stereo Investigator version 11.07.3 (MBF Bioscience, Williston, VT, USA) or IMARIS software version 9.9.1 (Oxford Instruments, Oxfordshire, UK).

### 5.13. Immunofluorescence Staining

The immunofluorescence staining procedure was conducted according to a previously described method [68]. Brain sections underwent three washes with 1× PBS and were treated with ice-cold methanol at −20 °C for 10 min to permeabilize the tissue. Following another three washes with 1× PBS, the sections were subjected to antigen retrieval by being heated in an 85 °C water bath for 10 min, with 10 mM citrate acid (pH 6.0). The sections were then blocked with a solution consisting of 2% BSA and 0.3% Triton™ X-100 in 1× PBS for 1 h. Primary antibodies, including those against CD68 (rabbit, 1:500, catalog no. 76437, Cell Signaling Technology, Danvers, MA, USA) and Iba-1 (mouse, 1:1000, catalog no. MABN92, Millipore, Burlington, MA, USA), were applied overnight at 4 °C. The sections were subsequently treated with secondary antibodies, anti-rabbit-647 (donkey, 1:200, catalog no. A31573, Thermo Fisher) and anti-mouse-555 (donkey, 1:200, catalog no. A31570, Thermo Fisher) for 2 h at 24 °C. Finally, the sections were mounted with DAPI solution (VECTASHIELD Antifade Mounting Medium with DAPI, catalog no. H-1200, Vector Laboratories, Newark, CA, USA) for nuclear staining. Panoramic Slide Scan or confocal microscopy (Confocal-A1R-MP, Nikon, Tokyo, Japan) was used to image the stained brain tissue samples. The intensity and number of amyloid plaques, as well as Iba-1 and CD68 staining, were analyzed using ImageJ version 1.53p (NIH, Bethesda, MD, USA) or IMARIS software version 9.9.1 (Oxford Instruments).

### 5.14. Western Blot

For protein expression analysis, hippocampus tissues were homogenized using a radioimmunoprecipitation assay solution containing a protease and phosphatase inhibitor cocktail. The Western blot protocol detailed in a previous reference was employed [69]. Briefly, protein samples extracted from the hippocampus tissues were quantified by means of the bicinchonic acid assay method, with protein quantities ranging from 10 to 100 μg. The appropriate amount of each target antibody was used to probe the quantified protein samples. Equal quantities of the quantified protein samples were loaded and mixed with 4× protein sample buffer before undergoing electrophoresis on pre-cast 4–12% polyacrylamide gels using an Invitrogen Western blot system (Invitrogen, Waltham, MA, USA) at 150 V for 40 min. Subsequently, the separated proteins were transferred onto polyvinylidene fluoride (catalog no. LC2002, Novex, Hochdorf, Switzerland) or nitrocellulose (catalog no. LC2000, Novex) membranes at 20 V for 2 h. Primary antibodies, including those against ACE1 (rabbit, 1:1000, catalog no. ab28311, Abcam, Cambridge, UK), ACE2 (rabbit, 1:1000, catalog no. ab15348, Abcam), IDE (mouse, 1:100, catalog no. SC-393887, Santa Cruz Biotechnology, Dallas, TX, USA), neprilysin (mouse, 1:100, catalog no. MA5-14050, Invitrogen), APP (mouse, 1:250, catalog no. 13-0200, Invitrogen), BACE1 (rabbit, 1:1000, catalog no. 5606, Cell Signaling Technology), PEN2 (rabbit, 1:1000, catalog no. 8598, Cell Signaling Technology), nicastrin (rabbit, 1:1000, catalog no. 5665, Cell Signaling Technology), LRP1 (rabbit, 1:1000, catalog no. 64099, Cell Signaling Technology), and RAGE (rabbit, 1:1000, catalog no. 42544, Cell Signaling Technology), were incubated with the membranes overnight at 4 °C. Following membrane washing using Tris-buffered saline with 0.3% Tween 20 Detergent (TBS-T), the secondary antibodies including IRD-800 anti-rabbit (donkey, 1:2000, catalog no. 926-32213, LI-COR, Lincoln, NE, USA) and IRD-800 anti-mouse (donkey, 1:5000, catalog no. 926-32212, LI-COR) were applied to the membranes for 2 h at 24 °C. After subsequent membrane washing, fluorescence signals were detected and analyzed utilizing Odyssey-CLx (LI-COR).

### 5.15. Statistical Analysis

All experimental analyzed data are presented as mean ± standard error of the mean. Statistical analysis was performed using Prism version 9 (GraphPad, San Diego, CA, USA), with one-way analysis of variance followed by Tukey’s HSD post hoc analysis to determine significant differences between groups. A *p*-value < 0.05 was considered statistically significant.

## Data Availability

The original data for this study are included in the paper and can be obtained from the corresponding author upon reasonable inquiry.

## Supplementary Materials

The following supporting information can be downloaded at https://www.mdpi.com/article/10.3390/ijms241914954/s1.
