# Supplementary material for: Transferrin-Conjugated Melittin-Loaded L-Arginine-Coated Iron Oxide Nanoparticles for Mitigating Beta-Amyloid Pathology of the 5XFAD Mouse Brain
Source: Int J Mol Sci. 2023 Oct 6;24(19):14954. doi: 10.3390/ijms241914954 (PMC10573775; doi:10.3390/ijms241914954)
Supplement: Supplementary file 1 [file ijms-24-14954-s001.zip › ijms-2621585-supplementary.pdf]

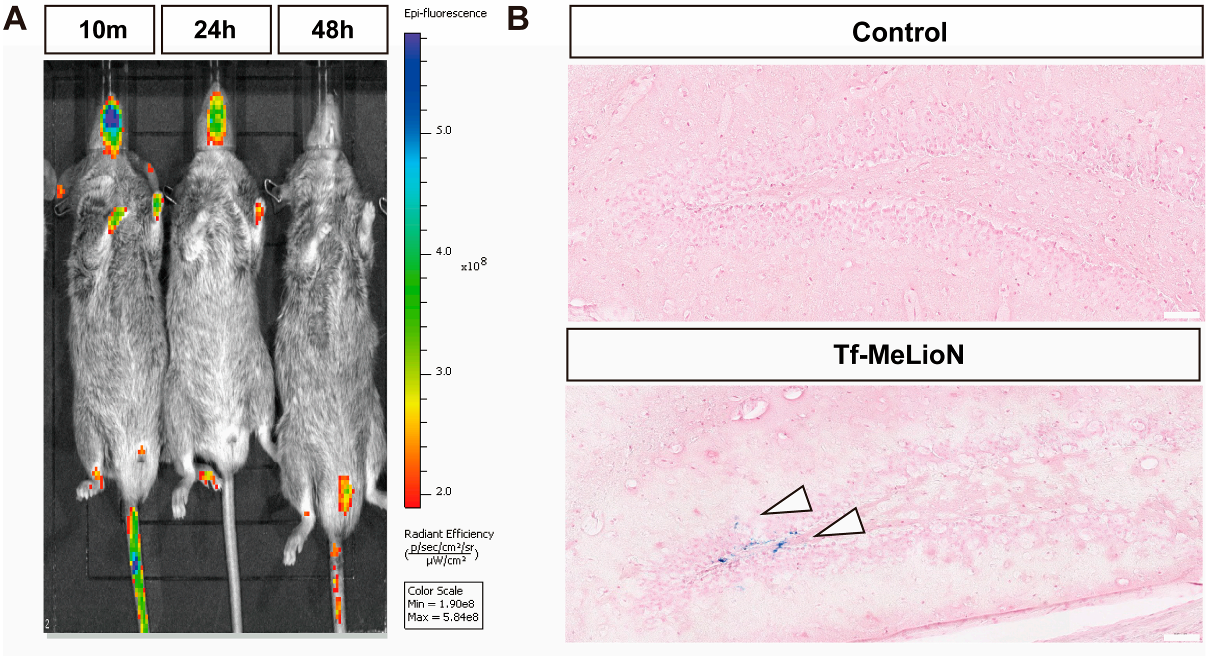

Supplementary Figure S1. Brain delivery of Tf-MeLioNs to 5XFAD mice. (A) Whole-mouse im aging was performed at various time-points (10 min, 24 h, and 48 h) using the IVIS® Lumina III system, following intravenous administration of Tf-MeLioNs. (B) Representative images of P russian Blue staining of 5XFAD mice tissues from the PBS- (control) and Tf-MeLioNs-administere d groups. Blue pigments (white arrow) in the DG of hippocampus indicate Tf-MeLioNs. Scale bar: 50 μm. Tf-MeLioNs, transferrin-conjugated melittin-loaded L-arginine-coated iron oxide na noparticles

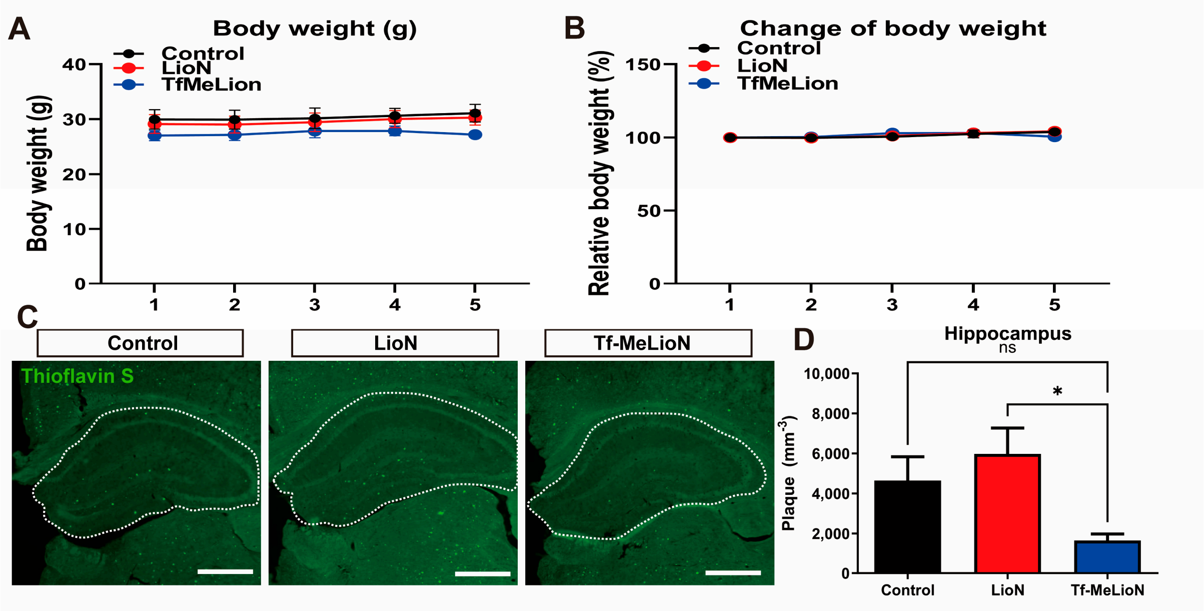

Supplementary Figure S2. Effects of Tf-MeLioN treatment on the number of amyloid plaques in the early stage of 5XFAD mice. Relative body weight change was observed and recorded weekly over five weeks **(A-B)**. Representative images of Thioflavin S staining of the hippocam pus tissues. Scale bar: 1000 μm (**C**). Quantification of the number of Thioflavin-S-positive plaq ues in the hippocampus (**D**). $^*p<0.05$ (one-way analysis of variance with Tukey's post hoc anal ysis). Data are presented as mean ± standard error of the mean. n=4-5 per group, for analyzin g the number of amyloid plaques. LioNs, L-arginine-coated iron oxide nanoparticles; Tf-MeLio Ns, transferrin-conjugated melittin-loaded LioNs
